# Label-free quantitative measurement of cardiovascular dynamics in a zebrafish embryo using frequency-comb-referenced-quantitative phase imaging

**DOI:** 10.1117/1.JBO.26.11.116004

**Published:** 2021-11-12

**Authors:** Jeeranan Boonruangkan, Hamid Farrokhi, Thazhe M. Rohith, Samuel Kwok, Tom J. Carney, Pei-Chen Su, Young-Jin Kim

**Affiliations:** a Nanyang Technological University, School of Mechanical and Aerospace Engineering, Singapore; b Nanyang Technological University, Lee Kong Chian, School of Medicine, Singapore; c Korea Advanced Institute of Science and Technology, Department of Mechanical Engineering, Daejeon, Republic of Korea

**Keywords:** quantitative phase imaging, frequency comb, cardiovascular dynamics, zebrafish, high-speed phase measurement

## Abstract

**Significance:** Real-time monitoring of the heart rate and blood flow is crucial for studying cardiovascular dysfunction, which leads to cardiovascular diseases.

**Aim:** This study aims at in-depth understanding of high-speed cardiovascular dynamics in a zebrafish embryo model for various biomedical applications via frequency-comb-referenced quantitative phase imaging (FCR-QPI).

**Approach:** Quantitative phase imaging (QPI) has emerged as a powerful technique in the field of biomedicine but has not been actively applied to the monitoring of circulatory/cardiovascular parameters, due to dynamic speckles and low frame rates. We demonstrate FCR-QPI to measure heart rate and blood flow in a zebrafish embryo. FCR-QPI utilizes a high-speed photodetector instead of a conventional camera, so it enables real-time monitoring of individual red blood cell (RBC) flow.

**Results:** The average velocity of zebrafish’s RBCs was measured from 192.5 to 608.8  μm/s at 24 to 28 hour-post-fertilization (hpf). In addition, the number of RBCs in a pulsatile blood flow was revealed to 16 cells/pulse at 48 hpf. The heart rates corresponded to 94 and 142 beats-per-minute at 24 and 48 hpf.

**Conclusions:** This approach will newly enable in-depth understanding of the cardiovascular dynamics in the zebrafish model and possible usage for drug discovery applications in biomedicine.

## 1 Introduction

The study of a cardiovascular system (including the heart, blood, and blood vessels) and its detailed functions is an important step toward developing cardiovascular drugs.^1^^,^^2^ In the field of biomedicine, drug discovery requires preclinical studies in an animal model before testing it in the human body. Zebrafish have been used as a good animal model for studying the human cardiovascular system, due to their optical transparency and fast development in their embryonic stage. Moreover, ∼70% of zebrafish genes are in common with human, and ∼84% of zebrafish genes are associated with human diseases.^3^^,^^4^ Various techniques have previously been adopted to provide quantitative assessments of the heart rate and blood flow in embryonic zebrafish. Bright-field microscopy with a high-speed camera is the most common tool for measuring the blood velocity; however, the image contrast has remained low due to the transparent nature of red blood cells (RBCs).^5^^,^^6^ Fluorescence microscopy has been introduced for a better image contrast with fluorescent labeling;^7^^–^^9^ however, it requires a transgenic modified zebrafish for imaging, which is phototoxic and time-consuming.

Label-free techniques have received much attention in the biological fields because the biological specimens can be monitored and investigated without any disturbance or alteration of their normal physiology. Fluorescence correlation spectroscopy has been developed as a minimally invasive fluorescence imaging technique that employs confocal microscopy to deduce the blood velocity from the autofluorescence fluctuation.^10^^,^^11^ The autofluorescence imaging of *in vivo* blood flow can be realized with two-photon microscopy;^12^ however, the autofluorescence from the blood cells is significantly low compared with the other nearby regions. The adaptive optics scanning laser ophthalmoscope has been used to measure the retinal blood velocity with a high lateral resolution; however, the detection speed is limited by the scanning rate.^13^ The optical vector field tomography [the optical equivalent of x-ray computed tomography (CT)] can provide a three-dimensional blood velocity map.^14^ Nevertheless, the sample rotation for data acquisition is troublesome and degrades the measurement speed. Recently, pupil-engineered localization microscopy, using an Airy-complementary kernel matching technique, has been used for high spatial and temporal resolutions for a high-volume blood flow analysis;^15^ however, this technique requires the injection of the fluorescent beads as a tracer, which is invasive. Quantitative phase imaging (QPI) was proposed to attain a minimally invasive quantitative measurement of biological specimens.^16^ Several studies have shown the successful use of QPI to measure cardiomyocyte dynamics over a wide field of view to the speed up to tens of Hz for various applications such as drug development.^17^^–^^20^

Doppler optical coherence tomography (DOCT) is one QPI technique that has been used to visualize the blood flow in a wider area.^21^^,^^22^ Digital particle image velocimetry was utilized for analyzing the velocity of blood flow and the individual RBC in DOCT. However, the RBC profile cannot be precisely resolved because the optical configuration does not support a high numerical aperture (NA) lens and is under a high-level laser speckle. In QPI, it is unavoidable to encounter the speckle effect, which could severely degrade the measurement precision.

In this study, we report that frequency-comb-referenced quantitative phase imaging (FCR-QPI) works as a simple, minimally invasive, and effective technique to measure the heart rate and blood flow of zebrafish embryos. The frequency comb (FC) has been used as a remarkable light source for QPI, which offers phase-coherent multiple wavelengths, suppressed speckle level, and high phase stability at a high measurement speed up to tens of kilohertz.^23^ Due to the speckle-suppression offered by this FCR-QPI technique, the RBCs flow in the zebrafish’s blood vessels could be monitored by both a slow CCD camera and a high-speed photodetector with minimum background speckle noise. The heart rate and blood flow of the zebrafish embryos at various ages [from 24 to 56 hour-post-fertilization (hpf)] were measured in real-time. The heart rate increased with the fish development from 94 beats-per-minute (bpm) at 24 hpf to 173 bpm at 50 hpf; then, it starts to decrease until 120 bpm at 56 hpf. The blood flow was measured as a form of the RBC velocity at 24 to 28 hpf ($V_{\text{mean}}=192.5$ to 608.8  μm/s). At 48 hpf, the pulsatile blood flow was monitored using a high-speed photodetector, which can reveal the individual RBC information (e.g., number of RBCs, diameter, and heartbeat) and the heartbeat development pattern as a vital sign of the heart’s health.

## 2 Frequency-Comb-Referenced QPI of Zebrafish Embryos

### 2.1 Frequency-Comb-Referenced Quantitative Phase Imaging

**Fig. 1:**
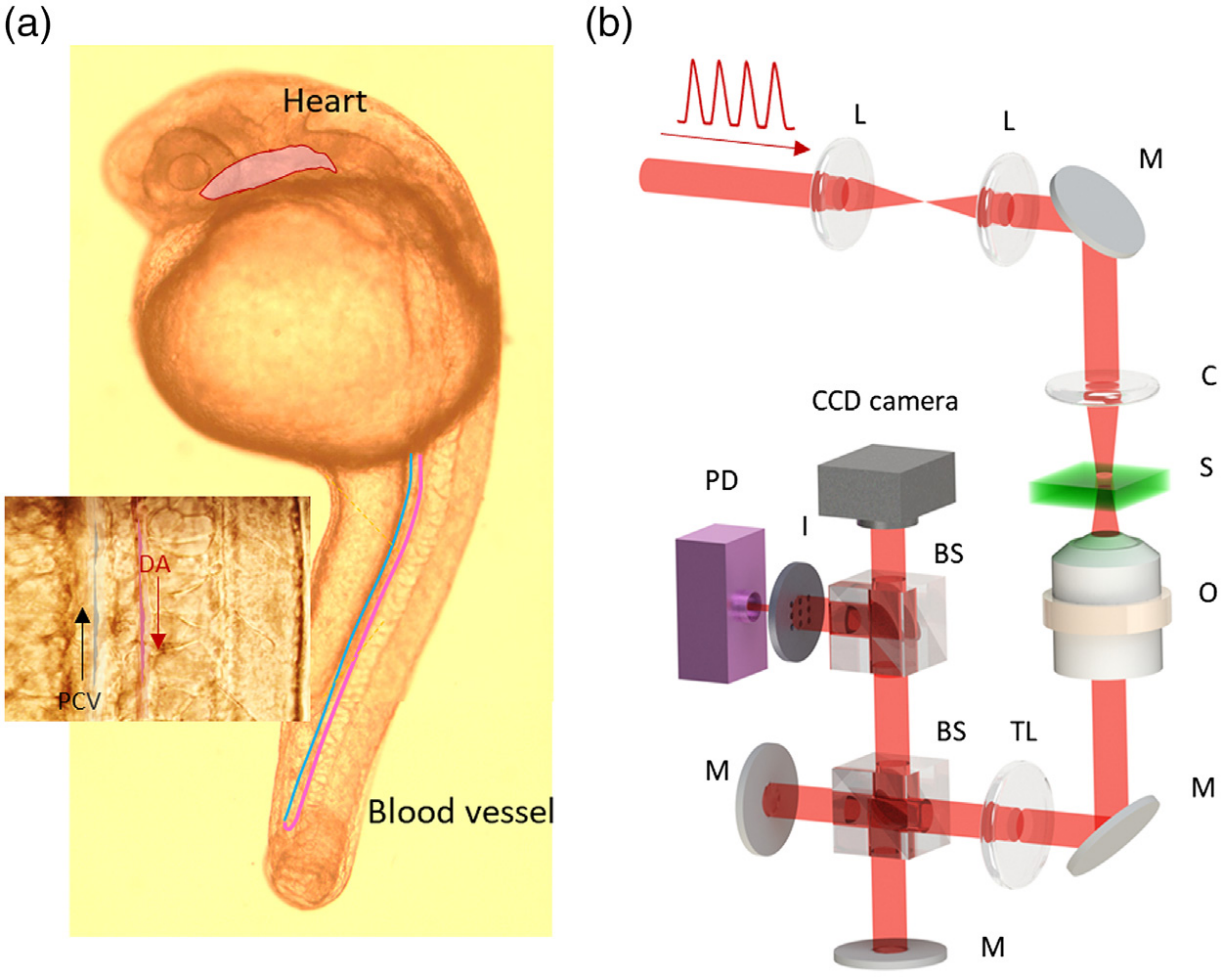
FCR-QPI. (a) Zebrafish embryo imaged under a WL microscope (M=10×). (b) System configuration of FCR-QPI: L, lens; M, mirror; C, collimator; O, objective lens; TL, tube lens; BS, beam splitter; I, iris; CCD, charge-coupled device; PD, photodetector.

A zebrafish at 24 hpf was fixed as a biosample in a microscope slide and imaged by a white-light (WL) bright-field microscope, which clearly showed the heart and blood vessels, as shown in Fig. 1(a). Main trunk vessels, which include the dorsal aorta (DA) and post-cardinal vein (PCV), were highlighted by red and blue lines, respectively. Figure 1(b) shows the optical configuration of the FCR-QPI. An Erbium-doped fiber FC emitted the ultrashort pulse train at a repetition rate of 250 MHz with a fundamental wavelength of 1550 nm. The wavelength was then converted to 780 nm by a nonlinear second harmonic generation. The beam was coupled into a photonic crystal fiber leading to spectral broadening to a supercontinuum ranging from 620 to 980 nm. The supercontinuum was delivered to illuminate the zebrafish’s heart and blood vessels located on the microscope. The light from the sample was collected by an objective lens (M=60, N.A.=0.7) and directed to the QPI unit, which is a modified version of Michelson’s interferometry based on off-axis and common-path design strategies.^24^ The QPI conﬁguration satisfies both off-axis and common-path geometry for high spatial data acquisition with high phase stability. When the illumination beam passes through the sample area, an unperturbed portion of the beam as a reference is carefully angled and superposed with the sample image. The modiﬁed Michelson-type QPI unit with controlled mirror angle matches the OPD for forming the low-coherence interferogram within the short temporal coherence length of the FC.^24^^–^^26^

Two types of detectors were utilized in this study: a CCD camera (DCU223, Thorlabs) and a Si-photodetector (PDA-36A, Thorlabs). The photodetector was used to monitor the high-speed dynamic motions of the RBCs. The CCD camera was used to locate the position of interest and provide spatial information of the heart and RBCs. For more details on FCR-QPI refer to Ref. ^27^.

### 2.2 Preparation of Zebrafish Embryo Samples

The zebrafish embryos were grown and maintained by standard procedures.^28^ The zebrafish embryos were derived from natural mating of the AB strain wild-type adults, housed in the LKC Medicine’s zebrafish facility at NTU-Singapore. The zebrafish housing, mating, and embryo manipulations were performed under the oversight of the NTU’s Institutional Animal Care and Use Committee (IACUC) under IACUC Protocol Number A18002 and complied with the National Advisory Committee for Laboratory Animal Research (NACLAR) Guidelines set out by the Agri-Food and Veterinary Authority (AVA) of Singapore. The embryos were collected and maintained in E3 media (5 mM NaCl, 0.17 mM KCl, 0.33 mM ${\mathrm{CaCl}}_{2}$, 0.33 mM ${\mathrm{MgSO}}_{4}$, $10^{-5}\%$ methylene blue). The embryos were dechorionated with watchmakers’ forceps and anesthetized with 0.02% Tricaine (buffered to pH=7.0). The embryos were added into 0.7% molten low melting agarose maintained at ∼42°C. The vacuum grease was used to draw a border around the edges of a standard microscope glass slide. The embryos were pipetted onto the glass slide inside the vacuum grease border and orientated using a fine gel loading tip. After the gel solidified, the vacuum grease-bordered area was flooded with E3 media, and a long glass coverslip was used to cover the mounted specimens (∼5 embryos/slide). Zebrafish aged 24 and 48 hpf were used in this study.

## 3 Measurement Results and Discussions

### 3.1 Comparative Study on Different QPI Light Sources

**Fig. 2:**
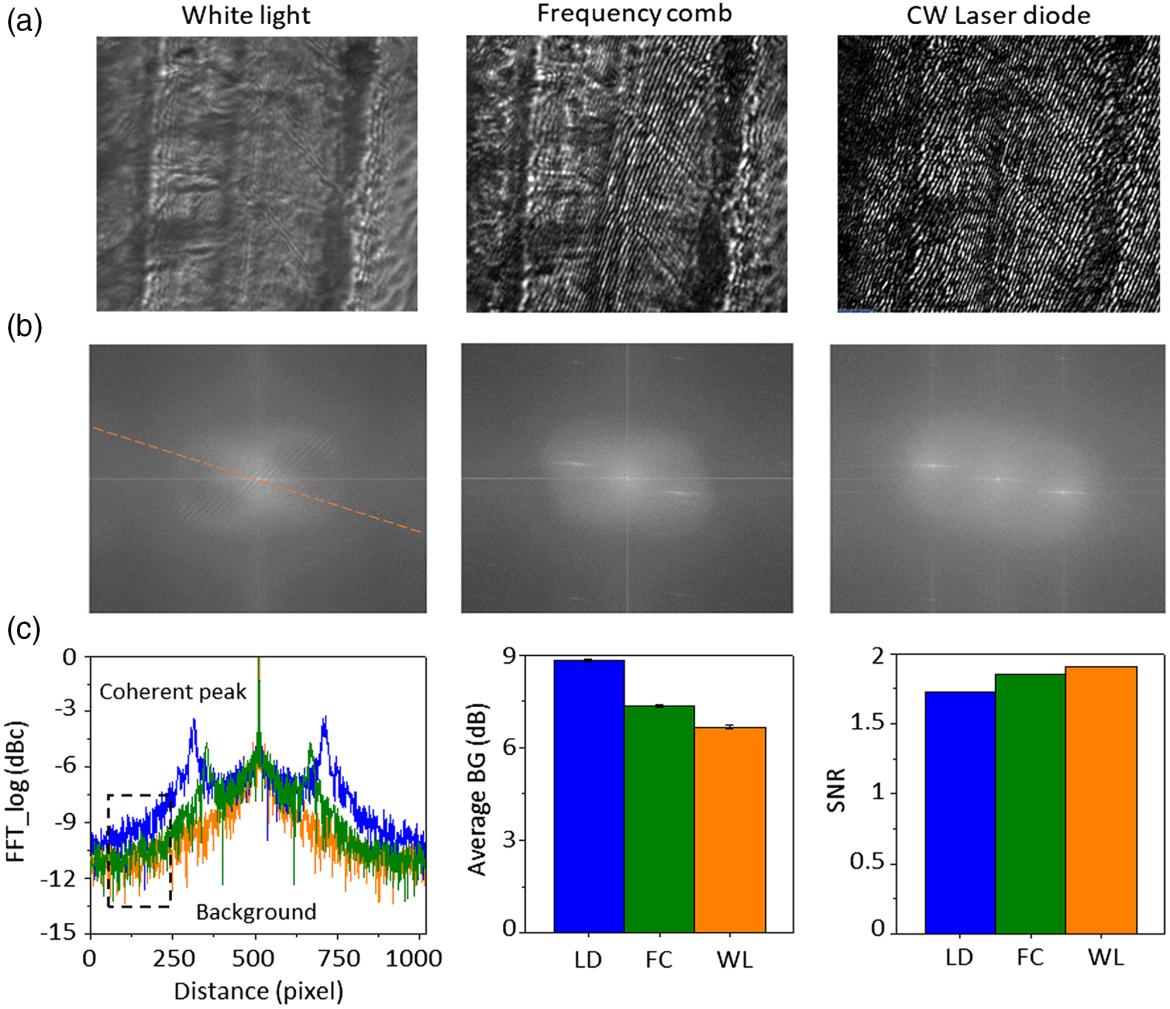
Evaluation of imaging performance of different light sources in QPI. (a) QPI images of zebrafish trunk illuminated by WL, FC, and continuous-wave LD. (b) FFT spectrums analyzed from the QPI images in (a). (c) Line extraction from the FFT spectrums of three light sources. (d) Average background (BG) noise and SNR, calculated in the Fourier domain.

We used three different light sources: WL, FC, and continuous-wave laser diode (LD) in the same QPI setup to evaluate their performances comparably. Figure 2(a) shows the QPI interferograms of the RBCs flowing through the vessel. To compare their imaging qualities, fast Fourier transform (FFT) analysis was performed on the images as shown in Fig. 2(b); the extracted FFT line profiles are shown in Fig. 2(c). Figure 2(c) clearly shows the side harmonics for FC and LD, which lack in WL FFT line. We used the (1) background speckle noise and (2) signal-to-noise ratio (SNR) for detailed analysis, as shown in Fig. 2(d). The background noise was suppressed from −9.90±0.05  dBc in LD to −10.65±0.05dBc and −11.34±0.06  dBc in FC and WL, respectively; these series of results imply that the background image gets cleaner with lower speckle levels. The QPI carrier frequency was used for the SNR analysis; the SNR analysis confirmed that the SNR of FC interferogram was higher than that of LD, implying the significant speckle noise suppression with the FC. The higher SNR leads to cleaner raw images, higher lateral resolution, and hence effective phase reconstruction. However, the SNR of the FC interferogram was slightly lower than that of WL (SNR = 1.85 for FC and 1.91 for WL). The relatively low (almost incoherent) QPI images by WL illumination cannot support phase reconstruction. Although the WL illumination has slightly higher SNR (DC/background signal level) comparing to the FC illumination, it can NOT support accurate and large FOV phase reconstruction due to lack of the harmonic components.

### 3.2 Interferogram and Dynamic Red Blood Cell Analysis

**Fig. 3:**
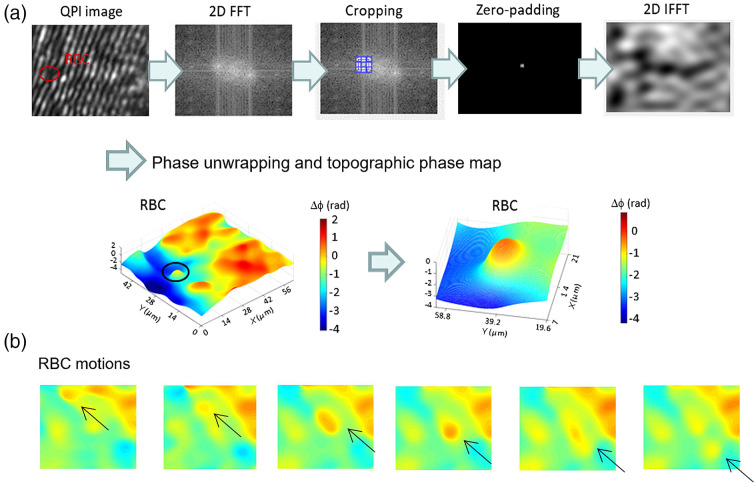
Phase reconstruction of an RBC from QPI interferogram. (a) Procedures for phase reconstruction of a single RBC. (b) Time-lapse phase maps of RBC flow in the blood vessel.

Phase reconstruction was performed by the FFT analysis. This process includes four modules: (1) 2D FFT, (2) cropping, shifting, and zero-padding, (3) 2D inverse FFT (IFFT), and (4) Goldstein’s phase unwrapping and phase map reconstruction modules. Following these procedures, the topographic phase map of RBCs in zebrafish’s blood vessel was reconstructed, as shown in Fig. 3(a). Because our approach is a single-shot approach, we could monitor the dynamic motion of RBCs with a higher temporal resolution (determined by the frame rate of a CCD). By performing phase reconstruction at each frame, time-lapse phase maps of RBC flowing in the blood vessel were obtained as shown in Fig. 3(b). The refractive index (RI) variation of the blood vessel can cause multiple scattering, which eventually results in higher speckle noise and hinder the successful phase reconstruction. Due to low temporal and high spatial coherence of the FC, which largely suppresses the speckle noise across the field and provides high visibility interferograms, we could easily attain the phase maps of individual RBCs inside the blood vessel with the suppressed coherent speckle noise level. We carefully select cropping size to include low- and high-frequency components for the phase reconstruction.

### 3.3 Heart Rate Measurement by FCR-QPI

**Fig. 4:**
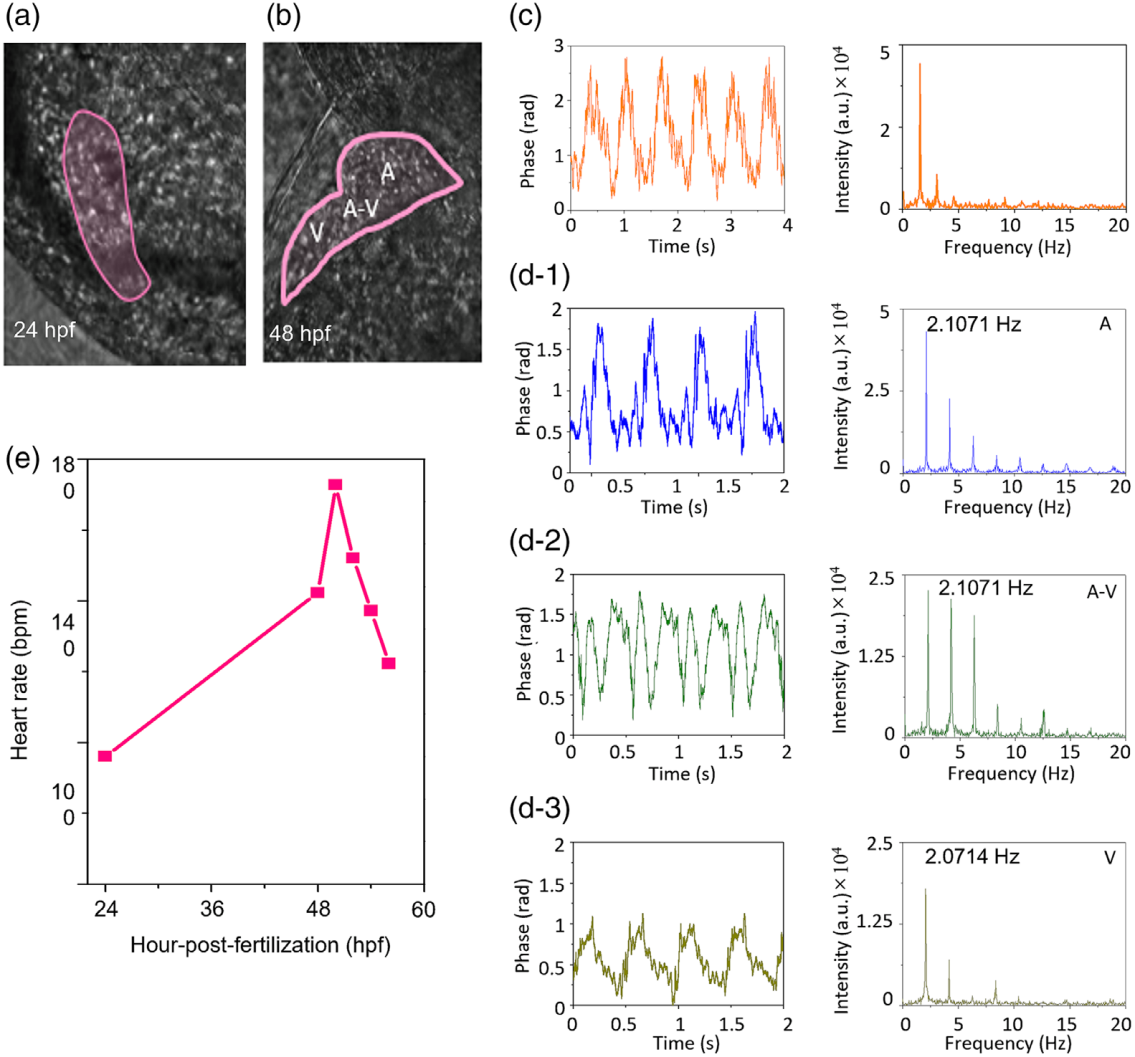
FCR-QPI for measuring the dynamic motions of zebrafish’s heart. Phase images of zebrafish’s heart at (a) 24 hpf and (b) 48 hpf. (c) Periodic signals of the heartbeat at 24 hpf analyzed in time and frequency domains. Periodic signals of the heartbeat at 48 hpf measured at (d-1) atrium (A), (d-2) A-V canal, and (d-3) ventricle (V). (e) Heart rate measured at various hpf.

FCR-QPI was employed to monitor the heart development and corresponding changes in the heart rates of zebrafish embryos from 24 hpf. A CCD camera was used first to obtain the overall morphology of the zebrafish’s heart, then the dynamic motions of the specific part of the heart were measured by a high-speed photodetector. Figures 4(a) and 4(b) show the QPI images of a heart tube at 24 hpf and a chambered heart at 48 hpf (pink shadow area). Figure 4(c) shows the measured phase modulation originating from the heart contraction and relaxation at 24 hpf. By performing the FFT, the frequency was determined to 1.56 Hz, corresponding to the heart rate of 94 bpm. The heartbeat in the zebrafish at 48 hpf was measured at different positions; the atrium (A), atrioventricular (A-V) canal, and ventricle (V) [see Figs. 4(d-1), 4(d-2), and 4(d-3)]. The period of the signal at the ventricle is set to be larger than the atrium because of the larger volume of the ventricle. At the A-V canal, both signals from the atrium and ventricle were superposed. We measured the heart rates in various fish ages as shown in Fig. 4(e). At an early stage of development, the heart rate increased from 94 bpm (24 hpf) to 173 bpm (50 hpf). Then, it began decreasing to 122 bpm at 56 hpf. It is noteworthy that the heart rate depends on not only the age but also the surrounding temperature; the temperature was set to 28°C in our study. These in-depth studies of the dynamic motion of zebrafish’s heart can be used to investigate the abnormality of heart rhythm (which is known as cardiac arrhythmia), exploration of new drugs, and testing of new therapeutics;^29^^–^^31^ for example, the heartbeat in a mutant zebrafish has been used to study the genetics related to the heart function.^32^

### 3.4 Blood Flow Measurement by FCR QPI

**Fig. 5:**
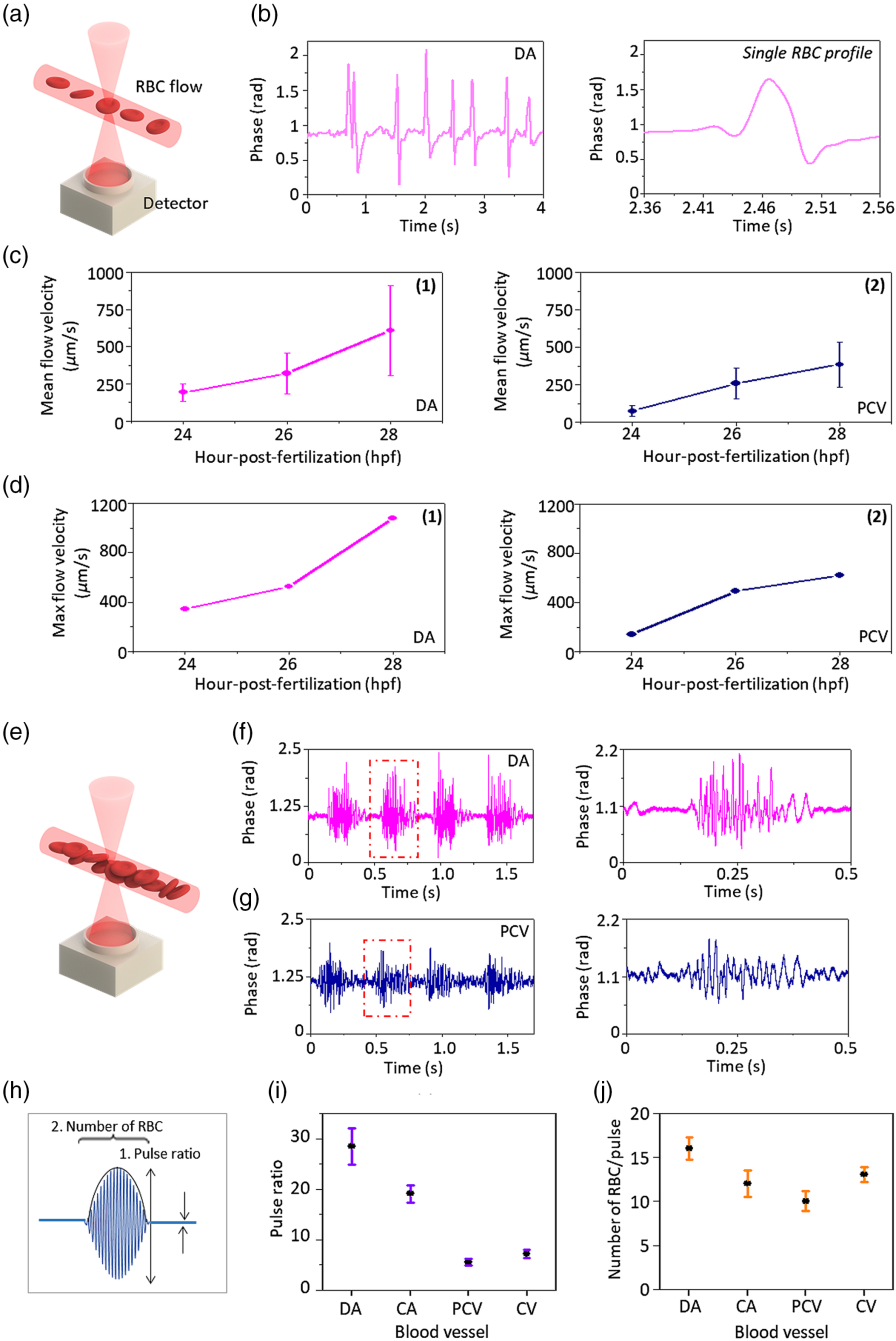
Blood flow measurement. (a) Schematic drawing of RBCs flowing in the blood vessels at 24 hpf. (b) Signal of RBCs flowing in the DA. (c) Mean RBC velocity in DA and PCV. (d) Maximum RBC velocity in DA and PCV. (e) Schematic drawing of RBCs flowing in the blood vessels at 48 hpf. Signal of pulsatile RBCs flowing in the (f) DA and (g) PCV. (h) Conceptual drawing of the number of RBCs and pulse ratio. (i) Number of RBCs and (j) pulse ratio in one blood pulse measured in DA, CA, PCV, and CV. DA, dorsal aorta; CA, caudal artery; PCV, post-cardinal vein; CV, caudal vein.

At 24 hpf, the blood flows in two main vessels: DA and PCV. The blood leaves the heart and travels in DA then returns to the heart via PCV at the tail region. At this stage, individual RBCs flowing in the blood vessels are detected, as shown in Fig. 5(a). The interference signal from RBCs flowing in DA was recorded using the photodetector and CCD camera while passing through the region of interest [see Fig. 5(a)]. The high-speed photodetector newly enabled the determination of the phase profile of individual RBCs, as shown in Fig. 5(b). The technique was to acquire the interferogram in the CCD camera first with many interference carrier fringes for FFT analysis. Then, we controlled the tilt angle of the interferometry mirror to lower down the number of fringes for PD measurement. This is to avoid the information being averaged out by many interferograms in a single PD readout. The point of interest is then chosen by the iris to obtain the phase information at a high-speed rate using PD. The programmable aperture-adjustable pinhole/iris is installed at the common path for the position match and coregistration of the CCD image and the photodetector signal. We acquired the same image using a beam splitter with the fully opened pinhole for CCD image registration, then gradually adjusting the pinhole size for the position match down to pixel level for PD signal registration.

We utilized the particle image velocimetry analysis for calculating the RBC velocity obtained by the CCD camera. The average RBC velocity increases as the fish develop from 24 to 28 hpf, and the velocity was higher in DA than PCV ($V_{\text{mean}}=192.5$ to 608.8  μm/s in the DA and 75.9 to 385.7  μm/s in the PCV) as shown in Figs. 5(c-1) and 5(c-2). The maximum RBC velocity in DA and PCV was calculated ($V_{\max\;}=345.2$ to 1079.9  μm/s in the DA and 139.5 to 618.8  μm/s in the PCV) as shown in Figs. 5(d-1) and 5(d-2). The in-depth understanding of vascular dynamics (i.e., blood velocity) can be beneficial for the investigation of endothelial dysfunctions that lead to vascular pathologies and diseases, including atherosclerosis, cardiovascular disease, and chronic kidney diseases.^33^^–^^36^ The blood flow in the zebrafish embryo can be used for the evaluation of vascular function.^37^ In addition, the initial blood flow in embryos plays an important role in cardiovascular development.^38^

At 48 hpf, the number of RBCs in the blood vessels and the velocity increase as shown in Fig. 5(e). At this stage, the chambered heart creates pulsatile blood flows, which have a large number of RBCs passing through the photodetector in a single heart pump, as shown in Figs. 5(f) and 5(g). Figures 5(f) and 5(g) show the detected interference signal of the pulsatile blood flow in the DA and PCV, which has several RBCs in a single magnified blood pulse. Four blood pulses correspond to the fourfold of heartbeat. In the DA, the blood pulses were compressed and traveled as an individual RBC package without a noticeable number of RBCs between different blood pulses. In contrast, while those individual blood pulses passing through PCV were expanded, so the spacing between them was filled in with several RBCs as shown in Fig. 5(g). For the quantitative analysis, the number of RBCs in a blood pulse and pulse ratios were calculated at main trunk vessels (DA and PCV) and caudal vessels [caudal artery (CA) and caudal vein (CV)]. CA and CV are the designated names of the DA and PCV in the tail portion. Figure 5(h) shows a conceptual drawing for calculating the number of RBCs and pulse ratio. The number of RBCs was obtained by counting the interference peaks of RBCs with aid of the cross-correlation method. The pulse ratio is the ratio of the highest peak (of the Gaussian fitting of the blood pulse) relative to the baseline. The average number of RBCs and average pulse ratio in a single blood pulse were highest in DA (N=16, pulse ratio = 28.5) and lowest in PCV (N=10, pulse ratio = 5.5) as shown in Figs. 5(i) and 5(j). This agrees with the fact that DA is located at the nearest distance from the heart, and PCV is farthest from the heart. Therefore, the blood exits the heart with a strong flow and gradually slows down at a long distance. The pulse ratio can reveal the heart’s performance for pumping the blood up. This study quantified the pulse ratio at different blood vessels, which could be useful in drug tests for relating their effects to heart-pumping.^39^ The time interval of individual RBC flown in the DA was 0.0152 s, which corresponds to 65.4 Hz.

### 3.5 Discussions

This series of experiments and evaluations confirmed that FCR-QPI could be employed to measure the cardiovascular dynamics in the zebrafish embryo, as shown in Figs. 4(a)–4(d) and 5(a)–5(j). FCR-QPI can provide the real-time and quantitative analysis of the RBCs in the blood vessels in a minimally invasive manner. This is highly beneficial in live-cell studies. With a higher visibility interferograms and single-shot detection capability of FCR-QPI, the phase of single RBC flow inside the blood vessel could be reconstructed as shown in Fig. 3(b). The RBC velocity of a 48 hpf zebrafish was measured at various locations within the heart using a high-speed photodetector, which could serve as the performance indicator.

The average number of RBCs in a single blood pulse was highest in DA, which was measured to be N=16. This was obtained by counting phase peaks at the PD measurement. We used the programmable iris to select a very small area in the middle of a vessel where RBCs pass could ensure the phase signals are mainly from RBCs. Meanwhile, the phase signal fluctuation from the vessel’s movement has significantly lower frequency compared to the RBC movement.

To suppress the unexpected noise and attain the clear RBC flow, we controlled the tilt angle of the reference mirror in the interferometry, which successfully filtered out the measurement noises and also did not average out the phase information.

To date, we have not validated our study against other methods, such as two-photon or multiphoton imaging. We will consider such validation processes in our next study.

It is worth noting that single file RBC movement in the vessels may not be a valid assumption at later development stages due to the enlarged size of vessels and higher pulse rate, which suggests high number and multilayer RBC passage through the point-of-interest and error in counting phase peaks. As we described, this method works only in the early stage. In the later development stage, we may consider extracting intensity signal fluctuation in-depth (i.e., such as A-line in OCT) to avoid the measurement error.

At the A-V canal, both signals from the atrium and ventricle were superposed. It is possible to separate the contractions with either PD temporal filtering or FFT/IFFT analysis.

Figure 4(e) is obtained from 3-fish measurements. The drops in heart rate after 48 hpf are not quite well understood at the moment. The new introduction of a high-speed photodetector enables us to overcome the traditional limit in temporal resolution with the universal unit of m/s.

## 4 Conclusion

We demonstrated the FCR-QPI as a minimally invasive and effective method for measuring the high-speed dynamic motion of zebrafish’s cardiovascular system at the cellular level. The heart rate of a zebrafish increased with the fish development up to 173 bpm at 50 hpf. The heart developed from the heart tube at 24 hpf to the chambered heart at 48 hpf, which was confirmed by the occurrence of the pulsatile blood flow at 48 hpf. The average blood flow velocity at 24 to 28 hpf was increased from 192.5 to 608.8  μm/s. By introducing the high-speed photodetector to the FCR-QPI, we could measure the high-speed RBC flow up to 65.4 Hz and extract the information on the number of RBCs at each blood pulse (16 cells/pulse) of a 48-hpf zebrafish. Minimally invasive, high-speed, and real-time monitoring of cellular RIs are highly requested for studying live zebrafish; for example, this study on the abnormality of cardiovascular dynamics in zebrafish can be used as a model for human vascular diseases. Therefore, FCR-QPI is expected to serve as a powerful tool for revealing out the details of human cardiac and vascular diseases.

## References

[1] Bournele D. Beis D., “Zebrafish models of cardiovascular disease,” Heart Failure Rev. 21, 803–813 (2016). 10.1007/s10741-016-9579-y 27503203

[2] Nguyen C. T. et al., “Zebrafish as a model for cardiovascular developments and disease,” Drug Discov. Today Dis. Models 5(3), 135–140 (2008). 10.1016/j.ddmod.2009.02.003 22275951 PMC3263402

[3] Howe K. et al., “The zebrafish reference genome sequence and its relationship to the human genome,” Nature 496, 498–503 (2013). 10.1038/nature12111 23594743 PMC3703927

[4] Kalueff A. V. Stewart A. M. Gerlai R., “Zebrafish as an emerging model for studying complex brain disorders,” Trends Pharmacol. Sci. 35, 63–75 (2014). TPHSDY 0165-6147 10.1016/j.tips.2013.12.002 24412421 PMC3913794

[5] Meissner R. et al., “*In vivo* vascular flow profiling combined with optical tweezers-based blood routing,” Proc. SPIE 10413, 104130H (2017). PSISDG 0277-786X 10.1117/12.2286117

[6] Anton H. et al., “Pulse propagation by a capacitive mechanism drives embryonic blood flow,” Development 140(21), 4426–4434 (2013). 10.1242/dev.096768 24089470

[7] Goetz J. G. et al., “Endothelial cilia mediate low flow sensing during zebrafish vascular development,” Cell Rep. 6(5), 799–808 (2014). 10.1016/j.celrep.2014.01.032 24561257

[r8] De Luca E. et al., “ZebraBeat: a flexible platform for the analysis of the cardiac rate in zebrafish embryos,” Sci. Rep. 4, 4898 (2014). SRCEC3 2045-2322 10.1038/srep04898

[9] Logan S. L. et al., “Automated high-throughput light-sheet fluorescence microscopy of larval zebrafish,” PLoS One 13(11), 0198705 (2018). POLNCL 1932-6203 10.1371/journal.pone.0198705 PMC6235235 30427839

[10] Shi X. et al., “Probing events with single molecule sensitivity in zebrafish and Drosophila embryos by fluorescence correlation spectroscopy,” Dev. Dyn. 238(12), 3156–3167 (2009). DEDYEI 1097-0177 10.1002/dvdy.22140 19882725

[11] Pan X. et al., “Characterization of flow direction in microchannels and zebrafish blood vessels by scanning fluorescence correlation spectroscopy,” J. Biomed. Opt. 12, 014034 (2007). JBOPFO 1083-3668 10.1117/1.2435173 17343509

[12] Zeng Y. et al., “Label-free *in vivo* flow cytometry in zebrafish using two-photon autofluorescence imaging,” Opt. Lett. 37(13), 2490–2492 (2012). OPLEDP 0146-9592 10.1364/OL.37.002490 22743431

[13] Palochak C. M. A. et al., “Retinal blood velocity and flow in early diabetes and diabetic retinopathy using adaptive optics scanning laser ophthalmoscopy,” J. Clin. Med. 8(8), 1165 (2019). 10.3390/jcm8081165 PMC6723736 31382617

[14] Du W. et al., “Optical projection tomography using a commercial microfluidic system,” Micromachines 11, 293 (2020). 10.3390/mi11030293 PMC7142877 32168806

[15] Zhou Y. et al., “High-speed extended-volume blood flow measurement using engineered point-spread function,” Biomed. Opt. Express 9(12), 6444–6454 (2018). BOEICL 2156-7085 10.1364/BOE.9.006444 31065441 PMC6490974

[16] Park Y. Depeursinge C. Popescu G., “Quantitative phase imaging in biomedicine,” Nat. Photonics 12, 578–589 (2018). NPAHBY 1749-4885 10.1038/s41566-018-0253-x

[17] Shaked N. T. et al., “Whole-cell-analysis of live cardiomyocytes using wide-field interferometric phase microscopy,” Biomed. Opt. Express 1, 706–719 (2010). BOEICL 2156-7085 10.1364/BOE.1.000706 21258502 PMC3018002

[r18] Rappaz B. et al., “Automated multi-parameter measurement of cardiomyocytes dynamics with digital holographic microscopy,” Opt. Express, 23, 13333–13347 (2015). OPEXFF 1094-4087 10.1364/OE.23.013333 26074583

[r19] Jaferzadeh K. et al., “Marker-free automatic quantification of drug-treated cardiomyocytes with digital holographic imaging,” ACS Photonics 7, 105–113 (2019). 10.1021/acsphotonics.9b01152

[20] Kemper B. et al., “Fast imaging of cardiomyocyte dynamics alterations after drug treatment utilizing quantitative phase digital holographic microscopy,” Proc. SPIE 11249, 112491S (2020). PSISDG 0277-786X 10.1117/12.2546255

[21] Rajan V. et al., “Review of methodological developments in laser Doppler flowmetry,” Lasers Med. Sci. 24, 269–283 (2009). 10.1007/s10103-007-0524-0 18236103

[22] Wang Y. et al., “*In vivo* total retinal blood flow measurement by Fourier domain Doppler optical coherence tomography,” J. Biomed. Opt. 12, 041215 (2007). JBOPFO 1083-3668 10.1117/1.2772871 17867804

[23] Boonruangkan J. et al., “Fast and sensitive quantitative phase imaging using a frequency comb,” in Sci. and Innov., CLEO USA (2019). 10.1364/CLEO_SI.2019.SM2H.3

[24] Baek Y. et al., “White-light quantitative phase imaging unit,” Opt. Express 24, 9308–9315 (2016). OPEXFF 1094-4087 10.1364/OE.24.009308 27137546

[r25] Kemper B. et al., “Simpliﬁed approach for quantitative digital holographic phase contrast imaging of living cells” J. Biomed. Opt. 16, 026014 (2011). JBOPFO 1083-3668 10.1117/1.3540674 21361698

[26] Girshovitz P Shaked N. T., “Compact and portable low-coherence interferometer with off-axis geometry for quantitative phase microscopy and nanoscopy” Opt. Express 21, 5701–5714 (2013). OPEXFF 1094-4087 10.1364/OE.21.005701 23482143

[27] Boonruangkan J. et al., “Coherence-tailored multiwavelength high-speed quantitative phase imaging with a high phase stability via a frequency comb,” Adv. Photonics Res. 2, 202000088 (2020). 10.1002/adpr.202000088

[28] Westerfield M., The Zebrafish Book. A Guide for the Laboratory Use of Zebrafish (Danio rerio), 4th ed., University of Oregon Press, Eugene (2000).

[29] Leong I. U. S. et al., “Zebrafish as a model for long QT syndrome: the evidence and the means of manipulating zebrafish gene expression,” Acta Physiol. 199(3), 257–276 (2010). 10.1111/j.1748-1716.2010.02111.x 20331541

[r30] Chico T. J. Ingham P. W. Crossman D. C., “Modeling cardiovascular disease in the zebrafish,” Trends Cardiovasc. Med. 18, 150–155 (2008). 10.1016/j.tcm.2008.04.002 18555188

[31] MacRae C. A., “Cardiac arrhythmia: *in vivo* screening in the zebrafish to overcome complexity in drug discovery,” Expert Opin. Drug Discovery 5(7), 619–632 (2010). 10.1517/17460441.2010.492826 PMC2935659 20835353

[32] Fink M. et al., “A new method for detection and quantification of heartbeat parameters in Drosophila, zebrafish, and embryonic mouse hearts” Biotechniques 46(2), 101–113 (2009). BTNQDO 0736-6205 10.2144/000113078 19317655 PMC2855226

[33] Gimbrone M. A. Jr., García-Cardeña G., “Endothelial cell dysfunction and the pathobiology of atherosclerosis,” Circul. Res. 118(4), 620–636 (2016). 10.1161/CIRCRESAHA.115.306301 PMC4762052 26892962

[r34] Aird W. C., “Endothelial cell heterogeneity and atherosclerosis,” Curr. Atheroscler. Rep. 8, 69–75 (2006). 10.1007/s11883-006-0067-z 16455017

[r35] Gutiérrez E. et al., “Endothelial dysfunction over the course of coronary artery disease,” Eur. Heart J. 34(41), 3175–3181 (2013). EHJODF 0195-668X 10.1093/eurheartj/eht351 24014385 PMC3814514

[36] Thambyrajah J. et al., “Abnormalities of endothelial function in patients with predialysis renal failure,” Heart 83, 205–209 (2000). 10.1136/heart.83.2.205 10648498 PMC1729306

[37] Watkins S. C. et al., “High resolution imaging of vascular function in zebrafish,” PLoS One 7(8), e44018 (2012). POLNCL 1932-6203 10.1371/journal.pone.0044018 22952858 PMC3431338

[38] Jones E. A., “The initiation of blood flow and flow induced events in early vascular development,” Semin. Cell Dev. Biol. 22, 1028–1035 (2011). SCDBFX 1084-9521 10.1016/j.semcdb.2011.09.020 22001248

[39] Milan D. J. et al., “Drugs that induce repolarization abnormalities cause bradycardia in zebrafish,” Circulation 107(10), 1355–1358 (2003). CIRCAZ 0009-7322 10.1161/01.CIR.0000061912.88753.87 12642353

